# Application of FISH based G2-PCC assay for the cytogenetic assessment of high radiation dose exposures: Potential implications for rapid triage biodosimetry

**DOI:** 10.1371/journal.pone.0312564

**Published:** 2024-10-25

**Authors:** Tammy L. Smith, Terri L. Ryan, Maria B. Escalona, Igor E. Shuryak, Adayabalam S. Balajee

**Affiliations:** 1 Cytogenetic Biodosimetry Laboratory, Radiation Emergency Assistance Center/Training Site, Oak Ridge Institute for Science and Education, Oak Ridge Associated Universities, Oak Ridge, Tennessee, United States of America; 2 Center for Radiological Research, Department of Radiation Oncology, Columbia University Medical Center, New York, New York, United States of America; King Saud University College of Pharmacy, SAUDI ARABIA

## Abstract

The main goal of this study is to test the utility of calyculin A induced G2-PCC assay as a biodosimetry triage tool for assessing a wide range of low and acute high radiation dose exposures of photons. Towards this initiative, chromosome aberrations induced by low and high doses of x-rays were evaluated and characterized in G2-prematurely condensed chromosomes (G2-PCCs) by fluorescence in situ hybridization (FISH) using human centromere and telomere specific PNA (peptide nucleic acid) probes. A dose dependent increase in the frequency of dicentric chromosomes was observed in the G2-PCCs up to 20 Gy of x-rays. The combined yields of dicentrics and rings in the G2-PCCs showed a clear dose dependency up to 20 Gy from 0.02/cell for 0.1 Gy to 14.98/cell for 20 Gy. Centric rings were observed more frequently than acentric ring chromosomes in the G2-PCCs at all the radiation doses from 1 Gy to 20 Gy. A head-to-head comparison was also performed by FISH on the yields of chromosome aberrations induced by different doses of x-rays (0 Gy -7.5 Gy) in colcemid arrested metaphase chromosomes and calyculin A induced G2-PCCs. In general, the frequencies of dicentrics, rings and acentric fragments were slightly higher in G2-PCCs than in colcemid arrested metaphase chromosomes at all the radiation doses, but the differences were not statistically significant. To reduce the turnaround time for absorbed radiation dose estimation, attempt was made to obtain G2-PCCs by reducing the culture time to 36 hrs. The absorbed doses estimated in x-rays irradiated (0,1,2 and 4 Gy) G2-PCCs after 36 hrs of culture were grossly like that of G2-PCCs and colcemid arrested metaphase chromosomes prepared after 48 hrs of culture. Our study indicates that the shortened version of calyculin A induced G2-PCC assay coupled with the FISH staining technique can serve as an effective triage biodosimetry tool for large-scale radiological/nuclear incidents.

## Introduction

Chromosome aberrations induced by genotoxic agents are traditionally studied in colcemid arrested metaphase cells in various animal and human cell model systems. One of the chromosome aberrations popularly known as dicentric chromosome is routinely analyzed in the peripheral blood lymphocytes of exposed humans for estimating the absorbed radiation dose. The dicentric chromosome assay (DCA) is considered as the gold standard for absorbed dose estimation and DCA is usually performed on stimulated peripheral blood lymphocytes at the metaphase stage of the first cell division. Application of DCA to radiation dose exposures higher than 5 Gy has generally proven problematic because the metaphase cells for dicentric chromosome analysis are highly restricted in number owing to an inherent radiosensitivity of human lymphocytes. These technical constraints have been overcome by preparation of prematurely condensed chromosomes either in G0/G1 phase by the cell fusion technique or in G2 phase by the use of protein phosphatase inhibitors such as calyculin A or okadaic acid [1–3]. The prematurely condensed chromosomes (PCCs) can be generated through fusion of G0 human lymphocytes with mitotic cells of Chinese Hamster Ovary (CHO) either by inactivated Sendai virus or by polyethylene glycol (PEG). G0 PCCs have been successfully utilized in several studies for the analysis of ionizing radiation induced chromosome aberrations [3–8]. Although the cell fusion technique is useful for the assessment of chromosome aberrations at high radiation doses [9, 10], clinical validation is still lacking for its widespread use in radiation dose estimation after accidental or incidental exposures in humans.

It is well established that infliction of DNA damage by physical and chemical agents including ionizing radiation triggers the activation of cell cycle checkpoints at G1, S and G2/M phases of the cell cycle in proliferating cells. Mitogenic stimulation of *ex vivo* irradiated lymphocytes in G0 leads to the accumulation of cells at G2/M phase and this G2/M phase accumulation occurs in a radiation dose dependent manner [11]. Therefore, the restricted availability of lymphocytes at the metaphase stage of mitosis following high radiation dose exposures is presumably due to the retarded progression of cells from G2 to mitosis. Since irradiated lymphocytes accumulate at the G2/M phase in a radiation dose dependent manner, efforts were made to generate G2-PCCs by using protein phosphatase inhibitors such as calyculin A and Okadaic acid that condense the G2 chromosomes prematurely [1, 2, 5, 8, 12–16].

As stated before, performing conventional DCA for radiation exposures higher than 5 Gy is somewhat difficult owing to either death or blockage of lymphocytes at G2 phase. Realizing the technical limitation of the conventional DCA, researchers started utilizing calyculin A induced G2-PCCs for analyzing the chromosome aberrations induced by high doses of radiation [10, 15–19]. High LET radiation induced damage was studied in G2-PCCs of ex vivo irradiated lymphocytes and in the blood samples of astronauts after short and long-term space mission [20]. A dose dependent increase in the formation of PCC-rings was observed in human lymphocytes irradiated with different doses of γ-rays (0 to 25 Gy) as well as varying proportions of γ-rays and neutrons [4]. Another study by Romero et al., (2013) utilized the PCC-ring assay for assessment of simulated high-dose partial body exposure in G2-PCCs performed with the initial fraction of irradiated cells that were serially diluted from 10% to 90%. Recently, caffeine-a non-specific inhibitor ATM/ATR kinases-was used to increase the fraction of calyculin A induced G2-PCC for the analysis of x-rays induced rings at high radiation doses [21]. Sun et al. [16] scored the frequencies of both total and hollow rings in G2-PCCs and determined that both gave a linear fit. Comparison of frequencies of rings in G2-PCCs and virus induced G1-PCCs revealed that both techniques can be effectively used for the biodosimetry analysis at high dose γ-rays exposures by scoring as low as 100 hollow rings.

Earlier studies have assessed the utility of PCC-rings for assessment of chromosome damage after exposure to different radiation qualities while other types of unstable chromosome aberrations (dicentrics and acentric chromosome fragments) were not fully evaluated and characterized for biodosimetry [15, 16, 18, 19]. Further, information on comparative evaluation of the yields of chromosome aberrations between colcemid arrested metaphase cells and calyculin A induced G2-PCCs is limited [22]. In this study, unstable chromosome aberrations (dicentrics, rings and acentric fragments) were systematically evaluated by FISH over a wide range of x-ray doses in both metaphase chromosomes and G2-PCCs. Utilization of centromeric and telomeric probes has greatly enabled us to categorize the nature of rings (acentric and centric) and acentric fragments (terminal, interstitial and compound) besides improving the overall accuracy of absorbed dose estimation by appropriate radiation dose response curves such as those developed in the present study for dicentrics and rings. Our study indicates that calyculin A induced G2-PCCs show higher yields of all the types of unstable chromosome aberrations than colcemid arrested metaphase chromosomes and that the analysis of G2-PCCs by FISH technique can serve as an effective biodosimetry tool which has the potential of detecting both low and acute high radiation dose exposures because of the improved resolution in aberration detection relative to that of the Giemsa staining technique.

## Materials and methods

### Blood collection

Collection of peripheral blood samples (10–20 ml) was performed from six healthy human volunteers (3 males and 3 females: 35–55 years of age) after the written consent of the donors in compliance with the Institutional Review Board (IRB) approved protocol (Oak Ridge Institute for Science and Education; IRB#: IRB00000547). The blood samples were utilized solely for the *in vitro* research purpose and no personal identifiable information was associated with any these study samples as per the HIPAA (Health Insurance Portability and Accountability Act) compliance.

### Irradiation

The x-ray irradiator RS-2000 (Rad Source, Suwanee, GA, USA) equipped with a 0.3 mm Copper filter irradiating at 160Kv at 25mA. There are 6 levels, but specimens are typically irradiated on levels 3,4,5 and 6 depending on the dose rates required for the experiment. In the current study, blood samples were irradiated at level 3 that delivered the dose rate of 2 Gy/min. The irradiator is annually calibrated by the manufacturer. To analyze radiation dose dependent induction of chromosome aberrations in G2-PCCs, aliquots of 1 ml of blood samples from a female donor were irradiated with various doses of x-rays (0 Gy to 20 Gy) using the irradiator facility at the University of Tennessee Knoxville (UTK). For the comparative analysis on the yields of chromosome aberrations in metaphase chromosomes and G2-PCCs, blood samples collected from 2 male donors were irradiated with different doses of x-rays (0, 0.5, 1, 3, 5 and 7.5 Gy). Aliquots of blood samples from a female donor were irradiated with different doses of x-rays (0, 1, 2 and 4 Gy) to compare the yields of dicentric chromosomes in metaphase chromosomes and G2-PCCs for absorbed dose estimation. Blood samples collected from two donors (one male and one female) were utilized for determining the optimal time of culture duration for the enrichment of G2-PCCs.

### Lymphocyte culture and chromosome preparation

Typically, cultures were set up in 15 ml conical tubes by mixing 500 μl from each of the mock and irradiated blood samples with 9.5 ml of complete growth medium (PBMAX, GIBCO) containing fetal bovine serum, growth factors, antibiotics and Phytohemagglutinin. The tubes were mixed and incubated at 37°C for 48 hrs. Colcemid (0.1 μg/ml) was added to the cultures for the last 4 hrs to prepare metaphase chromosomes. To prepare G2-PCCs, cultures were treated with a final concentration of 50 nm of calyculin A for the last 30 min. The harvesting procedure was essentially the same for both metaphase chromosomes and G2-PCCs. Cells, after the centrifugation step (1000 rpm for 10 min at ambient temperature), were treated with a prewarmed hypotonic solution (0.075 M KCl) for 18 min at 37°C followed by fixation in three changes of fixative (3-parts methanol and 1-part acetic acid). An aliquot of fixed cell suspension (30–40 μl) was placed at the center of acid cleaned glass slides and air dried.

### Fluorescence in situ hybridization (FISH) staining and image acquisition

FISH using Cy3-labeled centromere and fluorescein labeled telomere specific peptide nucleic acid (PNA) probes was performed to characterize the nature of unstable chromosome aberrations (dicentrics, centric rings, acentric rings, compound fragments, terminal, and interstitial fragments) in both colcemid arrested metaphase chromosomes and calyculin A induced G2-PCCs. The hybridization procedure was essentially the same as described by us before [9]. Briefly, the air-dried slides were first treated with 0.001% pepsin (Sigma-Aldrich, St. Louis, MO, USA) for 1–2 min followed by fixation for 10 min in a solution of 1% formaldehyde and 50 mM MgCl_2_ in 1X PBS (Phosphate Buffered Saline pH 7.0). Slides were covered with 40 μl of hybridization mixture containing the PNA probes, denatured at 80°C for 4 min followed by hybridization for 2 hrs at 37°C. After hybridization, the slides were washed for 5 min in 70% formamide and 2XSSC (Sodium Saline Citrate) followed by three washes of 5 min each in 4XSSC-0.05% Tween 20. After dehydration through ethanol series (50%, 70% and 100%, 2 min each), the slides were air dried and mounted 30 μl of DAPI (4’6-diamidino-2-phenylindole: Vector Laboratories, Burlingame, CA, USA) in an antifade solution. Images were captured using the metaphase finder algorithm (MetaSystems, Inc, Boston, MA, USA) under 10X objective followed by the image acquisition using 63X objective lens of the epifluorescence microscope (Carl Zeiss). Aberrations were analyzed manually in both metaphase chromosomes and G2-PCCs using the ISIS software.

### Analysis of chromosome aberrations

Unstable chromosome aberrations (dicentrics, rings and acentric fragments) were scored in both metaphases and calyculin A induced G2-PCCs after FISH staining. Rings were classified as centric and acentric based on the presence or absence of centromeric signal. Fragments were classified as interstitial (no telomeric signal), terminal (telomeric signal at one end) and compound (telomeric signal at both ends). The number of metaphases and G2-PCCs analyzed for different experiments are given in the respective Tables 1–5.

**Table 1:** Analysis of X-rays induced dicentric chromosomes in calyculin A induced G2-PCCs.

Dose (Gy)	Cells scored	Dic.	Distribution of dicentrics																				Yield ± SE	Var/Mean ± SE	U
			0	1	2	3	4	5	6	7	8	9	10	11	12	13	14	15	16	17	18	19			
**0**	**112**	**0**	**112**	**0**	**0**	**0**	**0**	**0**	**0**	**0**	**0**	**0**	**0**	**0**	**0**	**0**	**0**	**0**	**0**	**0**	**0**	**0**	**0**	**0**	**0**
**0.1**	**100**	**2**	**98**	**2**	**0**	**0**	**0**	**0**	**0**	**0**	**0**	**0**	**0**	**0**	**0**	**0**	**0**	**0**	**0**	**0**	**0**	**0**	**0.02 ± 0.01**	**0.99 ± 0.10**	**-0.10**
**0.25**	**135**	**7**	**128**	**7**	**0**	**0**	**0**	**0**	**0**	**0**	**0**	**0**	**0**	**0**	**0**	**0**	**0**	**0**	**0**	**0**	**0**	**0**	**0.05 ± 0.02**	**0.95± 0.11**	**-0.39**
**0.5**	**154**	**19**	**136**	**17**	**1**	**0**	**0**	**0**	**0**	**0**	**0**	**0**	**0**	**0**	**0**	**0**	**0**	**0**	**0**	**0**	**0**	**0**	**0.12 ± 0.02**	**0.98 ± 0.11**	**-0.10**
**0.75**	**282**	**53**	**234**	**43**	**5**	**0**	**0**	**0**	**0**	**0**	**0**	**0**	**0**	**0**	**0**	**0**	**0**	**0**	**0**	**0**	**0**	**0**	**0.18 ± 0.02**	**1.00 ± 0.08**	**0.05**
**1**	**220**	**84**	**154**	**51**	**12**	**3**	**0**	**0**	**0**	**0**	**0**	**0**	**0**	**0**	**0**	**0**	**0**	**0**	**0**	**0**	**0**	**0**	**0.38 ± 0.04**	**1.12 ± 0.09**	**1.30**
**2**	**188**	**133**	**94**	**64**	**23**	**5**	**2**	**0**	**0**	**0**	**0**	**0**	**0**	**0**	**0**	**0**	**0**	**0**	**0**	**0**	**0**	**0**	**0.70 ± 0.06**	**1.05 ± 0.10**	**0.48**
**3**	**119**	**161**	**30**	**41**	**30**	**13**	**4**	**1**	**0**	**0**	**0**	**0**	**0**	**0**	**0**	**0**	**0**	**0**	**0**	**0**	**0**	**0**	**1.35 ± 0.10**	**0.93 ± 0.13**	**-0.50**
**4**	**137**	**298**	**16**	**35**	**39**	**26**	**7**	**8**	**3**	**3**	**0**	**0**	**0**	**0**	**0**	**0**	**0**	**0**	**0**	**0**	**0**	**0**	**2.18 ± 0.14**	**1.16 ± 0.12**	**1.34**
**5**	**60**	**181**	**0**	**8**	**16**	**15**	**11**	**8**	**2**	**0**	**0**	**0**	**0**	**0**	**0**	**0**	**0**	**0**	**0**	**0**	**0**	**0**	**3.02 ± 0.22**	**0.61 ± 0.18**	**-2.11**
**7.5**	**37**	**210**	**0**	**1**	**0**	**3**	**5**	**8**	**9**	**6**	**1**	**4**	**0**	**0**	**0**	**0**	**0**	**0**	**0**	**0**	**0**	**0**	**5.68 ± 0.39**	**0.59 ± 0.23**	**-1.71**
**10**	**28**	**205**	**0**	**0**	**0**	**0**	**1**	**2**	**6**	**5**	**7**	**6**	**1**	**0**	**0**	**0**	**0**	**0**	**0**	**0**	**0**	**0**	**7.32 ± 0.51**	**0.30 ± 0.27**	**-2.56**
**15**	**25**	**241**	**0**	**0**	**0**	**0**	**0**	**1**	**1**	**2**	**4**	**5**	**4**	**2**	**3**	**2**	**0**	**1**	**0**	**0**	**0**	**0**	**9.64 ± 0.62**	**0.57 ± 0.28**	**-1.46**
**20**	**49**	**674**	**0**	**0**	**0**	**0**	**0**	**0**	**0**	**1**	**3**	**3**	**1**	**5**	**5**	**4**	**4**	**4**	**7**	**6**	**5**	**1**	**13.75 ± 0.53**	**0.76 ± 0.20**	**-1.15**

**Table 2:** Analysis of rings in calyculin A induced G2-PCCs after X-rays.

Dose (Gy)	Cells scored	Centric rings	Acentric rings	Total	Yield ± SE
0	112	0	0	0	0 ± 0
0.10	100	0	0	0	0 ± 0
0.25	135	0	0	0	0 ± 0
0.5	154	2	0	2	0.01 ± 0.01
0.75	282	5	0	5	0.01 ± 0.01
1	220	6	3	9	0.04 ± 0.01
2	188	11	6	17	0.09 ± 0.02
3	119	12	4	16	0.13 ± 0.03
4	137	19	9	28	0.20 ± 0.04
5	60	13	8	21	0.36 ± 0.07
7.5	37	14	5	19	0.48 ± 0.12
10	28	12	10	22	0.78 ± 0.17
15	25	22	17	39	1.56 ± 0.25
20	49	37	23	60	1.22 ± 0.15

**Table 3:** Comparative analysis of unstable chromosome aberrations detected in colcemid arrested metaphase chromosomes and calyculin A induced G2-PCCs by FISH.

**Metaphase chromosomes**												
Dose (Gy)	Cells	Dicentrics	**Yield ± SE**	Centric	Acentric	**Yield ± SE**	Compound	Terminal	Interstitial	**Yield + SE**	**Total**	**Yield ± SE**
0 (Donor 1)	250	0	**0**	0	0	**0**	2	1	0	**0.01 ± 0.00**	**3**	**0.01 ± 0.00**
0.5	250	20	**0.08 ± 0.02**	0	0	**0**	24	10	2	**0.14 ± 0.02**	**56**	**0.22 ± 0.03**
1	250	58	**0.23 ± 0.03**	1	0	**0.004 ± 0.00**	63	26	10	**0.39 ± 0.04**	**158**	**0.63 ± 0.05**
3	200	276	**1.38 ± 0.08**	6	1	**0.03 ± 0.01**	257	89	39	**1.93 ± 0.09**	**668**	**3.34 ± 0.13**
5	100	255	**2.55 ± 0.16**	9	4	**0.13 ± 0.03**	237	113	65	**4.15 ± 0.20**	**683**	**6.83 ± 0.26**
7.5	100	491	**4.91 ± 0.22**	19	5	**0.24 ± 0.05**	446	216	127	**7.89 ± 0.28**	**1304**	**13.00 ± 0.36**
0 (Donor 2)	221	0	**0**	0	0	**0**	2	1	1	**0.02 ± 0.01**	**4**	**0.02 ± 0.01**
0.5	164	10	**0.06 ± 0.02**	0	0	**0**	14	7	3	**0.14 ± 0.03**	**34**	**0.20 ± 0.01**
1	189	33	**0.17 ± 0.03**	3	0	**0.01 ± 0.01**	39	13	7	**0.31 ± 0.04**	**95**	**0.50 ± 0.05**
3	153	167	**1.09 ± 0.08**	3	2	**0.03 ± 0.01**	176	75	39	**1.90 ± 0.11**	**462**	**3.02 ± 0.14**
5	44	102	**2.32 ± 0.23**	4	1	**0.11 ± 0.05**	92	38	20	**3.41 ± 0.27**	**257**	**5.84 ± 0.36**
7.5	11	50	**4.54 ± 0.64**	3	0	**0.27 ± 0.15**	31	18	25	**6.73 ± 0.78**	**127**	**11.50 ± 1.02**
**G2-PCCs**												
Dose (Gy)	Cells	Dicentrics	**Yield ± SE**	Centric	Acentric	**Yield ± SE**	Compound	Terminal	Interstitial	**Yield ± SE**	**Total**	**Yield ± SE**
0 (Donor 1)	250	0	**0**	0	0	**0**	10	7	0	**0.07 ± 0.01**	**17**	**0.07 ± 0.01**
0.5	250	22	**0.08 ± 0.02**	0	0	**0**	29	29	8	**0.26 ± 0.03**	**88**	**0.35 ± 0.03**
1	250	56	**0.22 ± 0.03**	3	1	**0.01 ± 0.00**	86	61	18	**0.66 ± 0.05**	**225**	**0.90 ± 0.06**
3	200	281	**1.41 ± 0.08**	11	10	**0.10 ± 0.02**	291	181	91	**2.81 ± 0.12**	**865**	**4.33 ± 0.14**
5	100	321	**3.21 ± 0.18**	14	6	**0.20 ± 0.04**	288	163	92	**5.43 ± 0.23**	**884**	**8.84 ± 0.29**
7.5	100	576	**5.76 ± 0.24**	18	8	**0.26 ± 0.05**	530	310	181	**10.21 ± 0.32**	**1623**	**16.23± 0.40+**
0 (Donor 2)	250	0	**0**	0	0	**0**	4	4	6	**0.05 ± 0.01**	**14**	**0.05 ± 0.01**
0.5	250	22	**0.08 ± 0.02**	1	1	**0.008 ± 0.00**	47	28	2	**0.31 ± 0.03**	**101**	**0.40 ± 0.04**
1	250	61	**0.24 ± 0.03**	7	3	**0.04 ± 0.01**	74	34	9	**0.46 ± 0.04**	**188**	**0.75 ± 0.05**
3	200	247	**1.24 ± 0.08**	14	9	**0.11 ± 0.02**	279	144	47	**2.35 ± 0.11**	**740**	**3.70 ± 0.13**
5	100	268	**2.68 ± 0.64**	17	15	**0.32 ± 0.05**	260	163	77	**5.00 ± 0.22**	**794**	**7.94 ± 0.28**
7.5	100	500	**5.00 ± 0.22**	35	18	**0.53 ± 0.07**	535	254	123	**9.12 ± 0.0**	**1465**	**14.65± 0.38**

**Table 4:** Yields of dicentric chromosomes at two different fixation times and four radiation doses in calyculin A induced G2-PCCs.

Dose (Gy)	Cal A (36 hrs)			Cal A (48 hrs)			Colcemid (48 hrs)		
	Cells	Dicentrics	Mean ± SEM	Cells	Dicentrics	Mean ± SEM	Cells	Dicentrics	Mean ± SEM
0	102	0	0	50	1	**0.02 ± 0.02**	500	0	**0**
1	50	10	**0.20 ± 0.06**	50	9	**0.18 ± 0.06**	100	20	**0.20 ± 0.05**
2	50	22	**0.44 ± 0.09**	50	30	**0.60 ± 0.11**	100	42	**0.42 ± 0.07**
4	20	31	**1.55 ± 0.28**	17	31	**1.82 ± 0.33**	100	185	**1.85 ± 0.23**

**Table 5:** Comparison of radiation dose estimates in calyculin A induced G2-PCCs and in colcemid arrested metaphase chromosomes.

Estimated dose (Gy)						
Delivered dose (Gy)	Cal A- 36 hr	LCL-UCL (95% CI)	Cal A- 48 hr	LCL-UCL (95% CI)	Col- 48 hr	LCL-UCL (95% CI)
0	0	0	0.11	0–0.11	0	0
1	1.02 ± 0.20	0.60–1.53	0.95 ± 0.21	0.53–1.46	1.02 ± 0.15	0.72–1.37
2	1.71 ± 0.18	1.27–2.20	2.07 ± 0.16	1.62–2.55	1.66 ± 0.14	1.35–2.0
3	3.58 ± 0.37	2.87–4.35	3.92 ± 0.41	3.15–4.76	3.95 ± 0.20	3.64–4.28

### Determination of optimal culture time for the enrichment of G2-PCCs

To determine the feasibility of reducing the culture time for obtaining G2-PCCs, calyculin A was added to the unirradiated cultures at different times after culture initiation (28, 30, 32, 34, 36 and 40 hrs). Calyculin A treated cells were harvested and the percentage of cells in different phases (G1, S and G2) were identified based on chromosome morphology: G1-PCCs with single chromatid, S-PCCs- fragmented and G2-PCCs with two chromatids [21].

### Estimation of absorbed radiation dose in G2-PCCs obtained at two different fixation times

Mock and irradiated lymphocyte (1, 2 and 4 Gy) cultures were harvested at 36 hrs and 48 hrs following the treatment with 50 nm calyculin A for 30 min. The estimated doses for G2-PCCs were compared with colcemid arrested metaphases obtained after 48 hrs of culture. A standard calibration curve generated for X-rays using the FISH based detection of dicentrics [Y = 0.008 ± 0.004 + 0.088 ± 0.022* D+ 0.095 ± 0.009)*D^2^ where Y is the yield of dicentrics, and D is the radiation dose] was used for dose estimation using the Dose Estimate algorithm.

### Statistical analysis

Statistical analysis (Yield of aberrations ± SE, Variance/Mean ± SE and U test for under or overdispersion of aberrations) was performed using the Dose Estimate software. Correlation coefficient analysis was performed for comparing the yields of chromosome aberrations in colcemid arrested metaphase chromosomes and calyculin A induced G2-PCCs. Multivariate Response Generalized Linear Models (MGLM; https://cran.r-project.org/web/package/MGLM/index.html) R package was used to determine whether there were differences in the yields of chromosome aberrations (dicentrics, rings and acentric fragments) between calyculin A induced G2-PCCs and colcemid arrested metaphase chromosomes. The modeling involved linear quadratic (LQ) functions of radiation dose for comparison between acentric rings and centric rings as well as among different types of fragments (compound, interstitial and terminal). For this purpose, Wald tests were done on the estimated alpha and beta parameters and their standard errors to verify whether alpha beta values were statistically different for rings (acentric vs centric rings) and among different types of acentric fragments.

## Results

### FISH analysis of unstable chromosome aberrations in G2-PCCs

**Fig 1:**
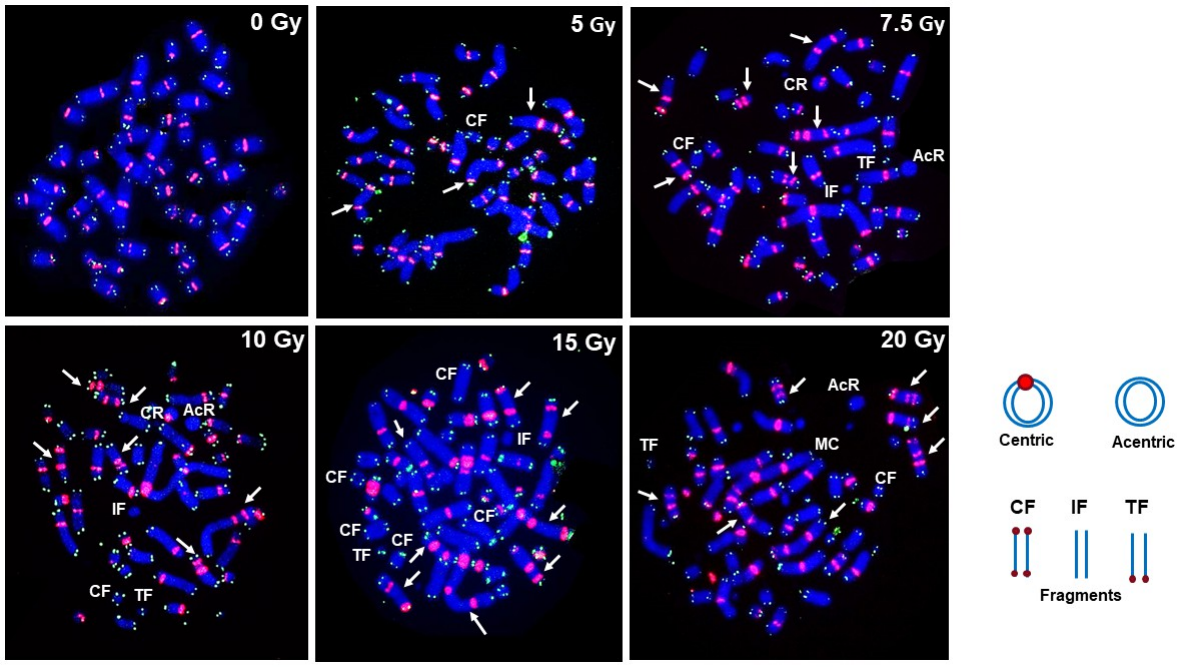
Representative pictures of calyculin A induced G2-PCCs prepared from mock and irradiated lymphocytes with different doses of x-rays. Dicentric and multicentric chromosomes are indicated by arrows. CF-Compound fragment, TF- Terminal fragment, IF-Interstitial fragment, MC-Multicentric chromosomes, AcR-Acentric ring and CR-Centric ring.

**Fig 2:**
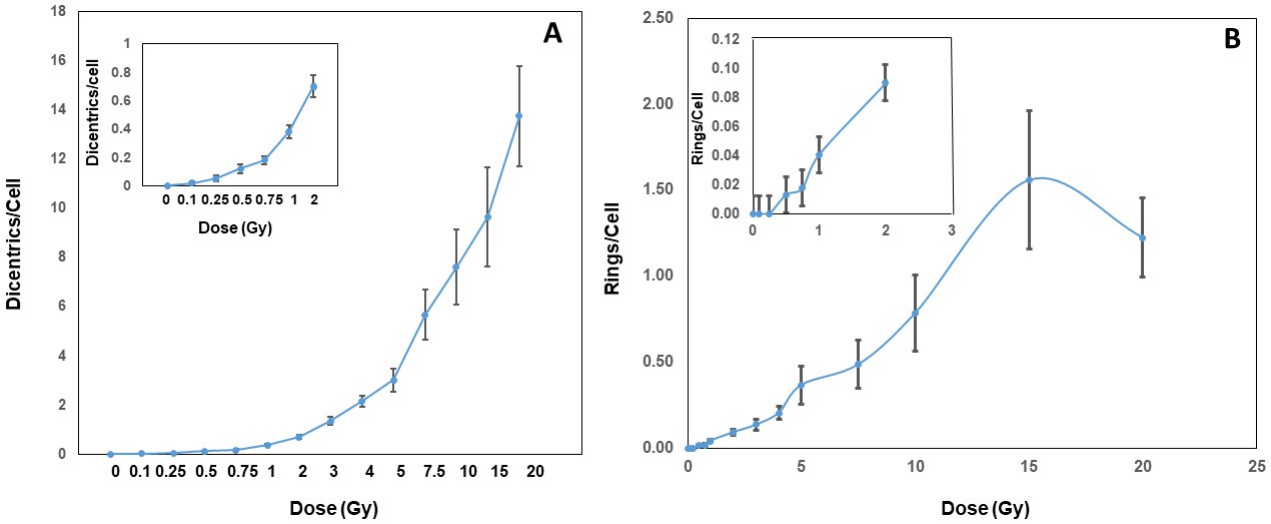
Frequencies of dicentric (A) and ring chromosomes (B) induced by different doses of x-rays in calyculin A induced G2-PCCs. Yields of dicentric, and ring chromosomes induced by 0.1 to 2 Gy of x-rays are shown in the inserts. Error bars indicate SD of the mean.

**Fig 3:**
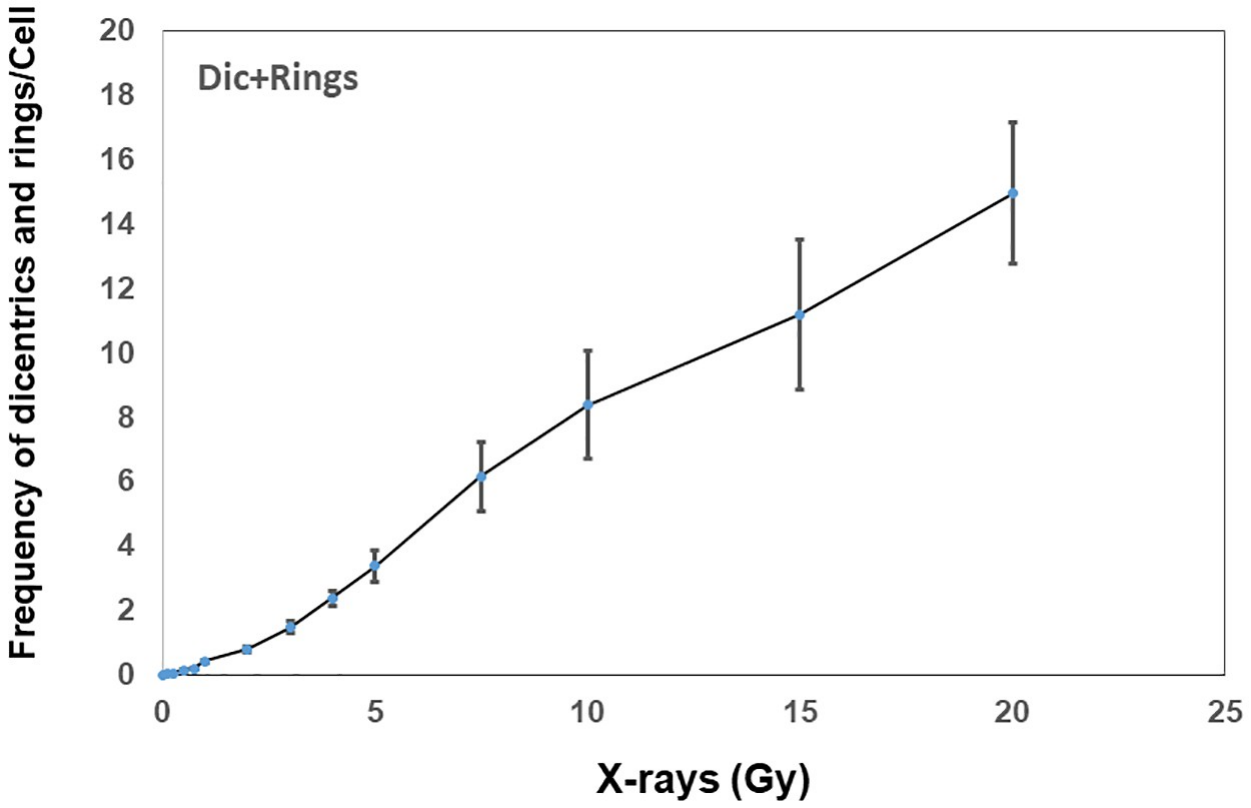
Combined yields of dicentric chromosomes and ring chromosomes in calyculin A induced G2-PCCs after different doses of x-rays are shown. Error bars indicate SD of the mean.

Representative pictures of calyculin A induced G2-PCCs of mock and X-rays treated lymphocytes that were hybridized with centromere and telomere specific PNA probes are shown in Fig 1. Data on the frequency and distribution of dicentric chromosomes observed in G2-PCCs after exposure to various doses of X-rays (0–20 Gy) are summarized in Table 1. The main goal of this manuscript was to determine the utility of G2-PCC assay over a wide range of radiation doses (0–20 Gy) to provide a rapid medical triage by scoring fewer cells (typically 100–200 cells for doses up to 4 Gy and 50 cells or less for doses higher than 4 Gy) to estimate the absorbed radiation doses. Scoring 500 cells or more will considerably increase the turnaround time for dose estimation thereby causing a substantial delay for triage. As expected multicentric chromosomes (tricentric, quadricentric, pentacentric, hexacentric etc.) were observed at doses higher than 3 Gy and cells with more than 10 dicentric chromosomes were observed at doses higher than 10 Gy. The number of dicentric chromosomes in multicentric chromosomes (three or more centromeres) was estimated by the total number of centromeres minus 1. For instance, one tricentric chromosomes was counted as two dicentric chromosomes (3–1 = 2). The frequency of dicentric chromosome detected by centromere/telomere FISH was 0.02 ± 0.01/cell for 0.1 Gy and 13.75 ± 2.03 /cell for 20 Gy. The calibration coefficients determined by CABAS (Chromosome Aberration Analysis Software [23]) are Y = 0 + 0.021 ± 0.002*D + 0.387 ± 0.017*D^2^ where Y is the yield of dicentrics, and D is the radiation dose. We next analyzed the distribution of dicentric chromosomes/cell as a function of radiation dose using “fitdistrplus” R package and the results showed that the dicentric distribution for different doses were consistent with Poisson even at radiation doses exceeding 10 Gy. The frequencies of dicentric chromosomes and ring chromosomes (centric and acentric) detected as a function of radiation dose are shown in Fig 2A and 2B and the combined frequencies of dicentrics and rings detected for different doses of x-rays (0–20 Gy) are shown in Fig 3.

**Fig 4:**
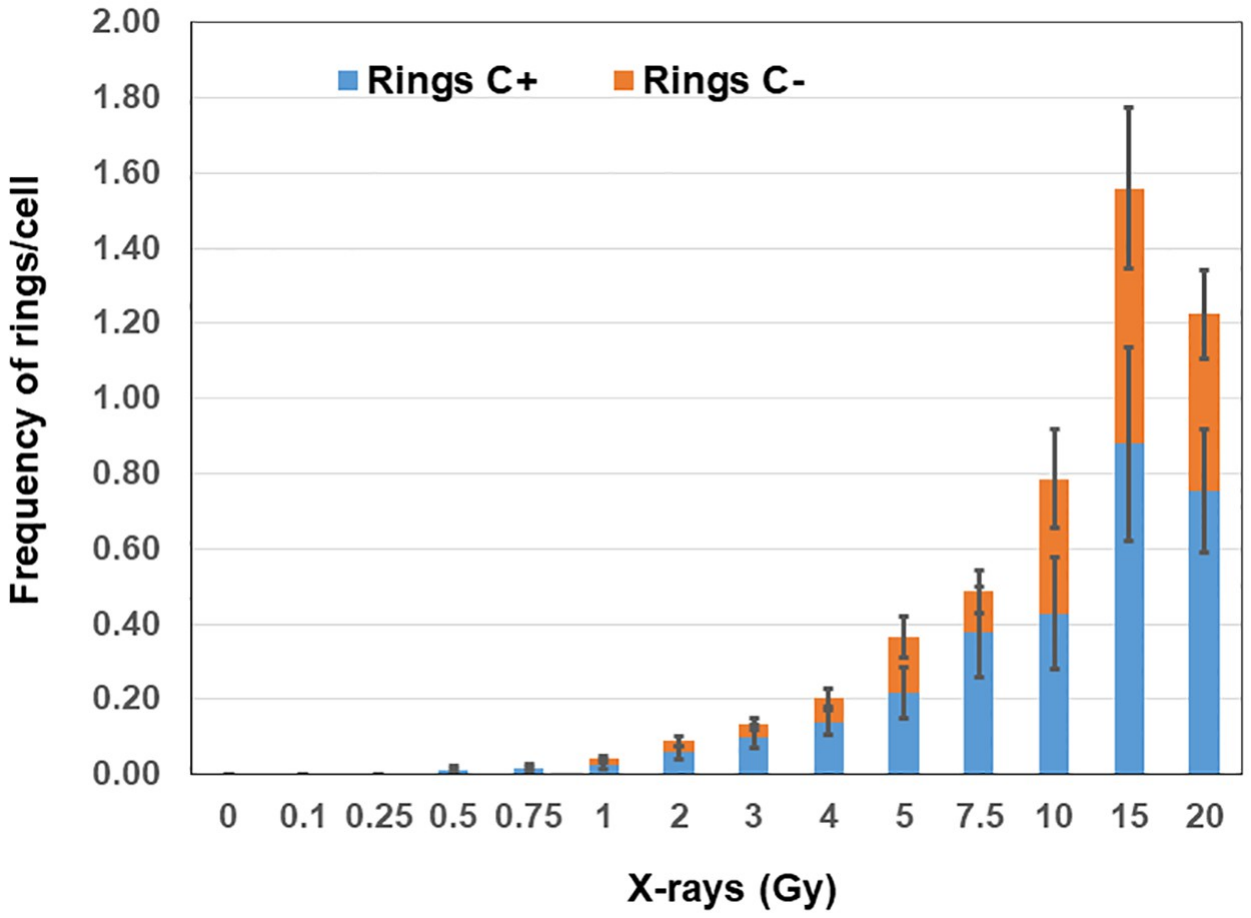
Frequencies x-rays induced centromere positive and negative ring chromosomes observed in G2-PCCs are shown. Error bars indicate SD of the mean.

The total number of rings (centric and acentric) detected in the G2-PCCs of mock and irradiated lymphocytes are shown in Table 2 and Fig 2B. Centric rings (centromere positive) occurred much more frequently than acentric rings at all the radiation doses and the centric rings constituted 60–75% of the total rings at all radiation doses (Fig 4). The calibration coefficients determined by the regression analysis of rings using the CABAS are Y = 0 + 0.001*D + 0.051*D^2^. The formation of rings showed a dose dependent increase up to 15 Gy with a slight decline at 20 Gy. However, the combined yields of dicentrics and rings showed a dose dependent increase from 0.1 Gy to 20 Gy (Fig 3) and the R^2^ values for linearity were found to be 0.978 for dicentrics and 0.858 for rings. The Multivariate Response Generalized Linear Models (MGLM) R package was next used to analyze the two correlated outcomes, centric rings and acentric rings, and the modelling involved the fitting Linear quadratic (LQ) functions of radiation dose. The Wald tests performed on the estimated alpha and beta parameters with their standard errors were used to determine whether alpha and beta values were different for centric and acentric rings. The *p*-values obtained from these tests were 0.35 for alpha and 0.52 for beta suggesting that the differences in yields of acentric and centric rings were not statistically significant.

### Comparative analysis of chromosome aberrations in metaphase chromosomes and G2-PCCs

**Fig 5:**
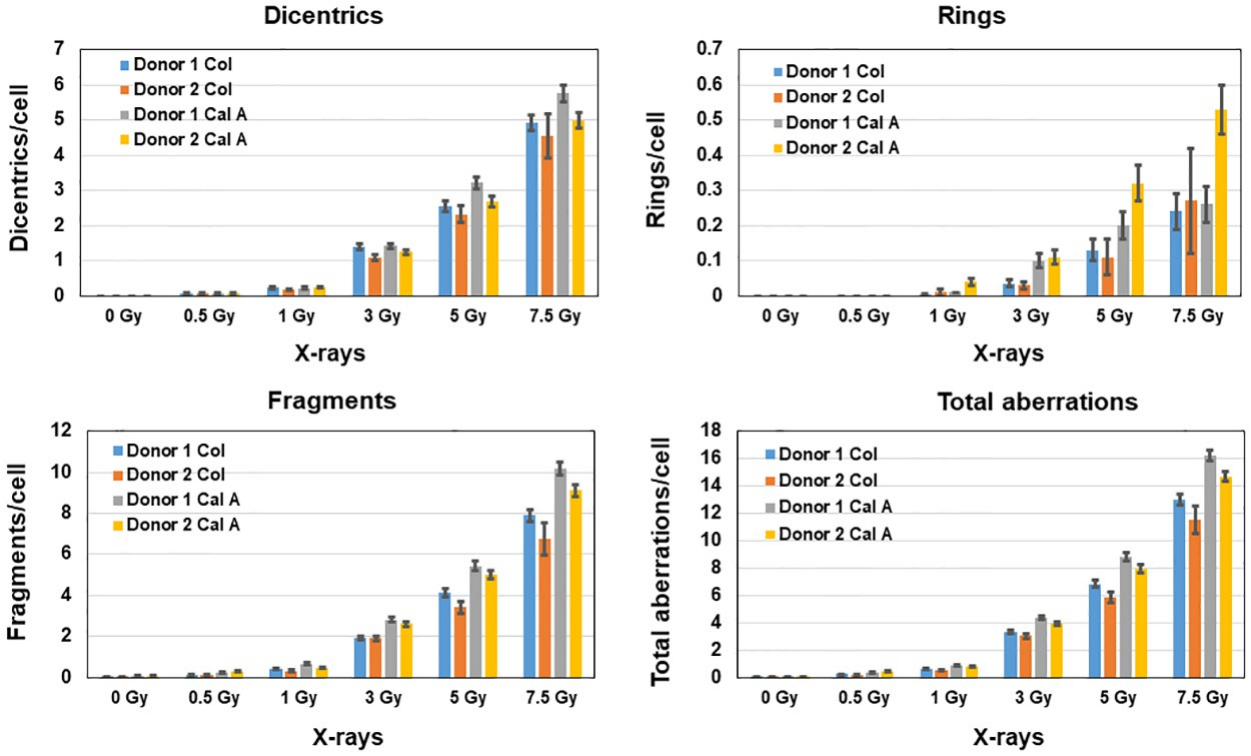
Comparative analysis on the yields of x-rays induced unstable chromosome aberrations (dicentrics, rings and fragments) in colcemid arrested metaphase chromosomes (Col) and calyculin A (Cal A) induced G2-PCCs performed in the blood samples of two donors are shown. Error bars indicate SEM.

To determine whether radiation induced chromosome aberration yields are different in metaphase chromosomes and calyculin A induced G2-PCCs, blood samples collected from 2 male donors were irradiated with various doses of x-rays (0.5, 1, 3, 5 and 7.5 Gy) and the cultures were set up separately for the preparation of colcemid arrested metaphase chromosomes and calyculin A induced G2-PCCs. FISH was used with centromere and telomere specific PNA probes to evaluate and characterize the unstable aberrations (dicentrics, rings and fragments). Rings were classified as either centric or acentric based on the presence or absence of centromeric signal. Terminal, interstitial and compound chromosome fragments were classified based on the presence or absence of telomeric signal. Data on the yields of aberrations observed in the metaphase chromosomes and G2-PCCs of the two donor samples are summarized in Table 3. Frequencies of dicentric chromosomes, rings (centric and acentric) and fragments (compound, terminal and interstitial) observed in metaphase chromosomes and G2-PCCs of the two donor samples are shown in Fig 5. In general, yields of dicentrics, rings and acentric fragments in both donor samples were higher in the G2-PCCs than in the metaphase chromosomes despite variations between the samples of two donors. Except for rings, both dicentric chromosomes and fragments were consistently reduced at all the radiation doses in both metaphase chromosomes and G2-PCCs of donor 2 sample. The dicentric frequencies per cell were similar up to 3 Gy in both colcemid arrested metaphase chromosomes and G2-PCCs, but G2-PCCs showed slightly higher frequencies of dicentrics than metaphase chromosomes at 5 Gy and 7.5 Gy exposures in both donors (Table 3). As stated before, centric rings were detected much more frequently than acentric rings in the metaphase chromosomes and G2-PCCs of both donors and the frequencies of rings were higher in G2-PCCs of both donor samples (Table 3; Fig 5). The frequency of rings observed in the G2-PCCs of donor 2 was 1.6–2 folds higher than that of donor 1 at 5 Gy and 7.5 Gy exposures respectively.

FISH using centromere and telomere specific probes enabled us to precisely identify the nature of acentric fragments based on the presence or absence of telomeric signal at one or both ends of the chromosome fragments. In general, compound fragments with telomeric signal at both ends (presumably resulting from the fusion of terminal fragments of one or two chromosomes) constituted more than 50% of the total fragments in the irradiated samples [with the exception of 7.5 Gy sample of donor 2 in the metaphase chromosomes (41.89%) and 0.5 Gy sample of donor 1 in G2-PCCs (43.93%)] followed by terminal fragments (telomeric signal at one end) and interstitial fragments albeit variations between two donors in both metaphase chromosomes and G2-PCCs. Like dicentrics and rings, frequency of total fragments observed in both metaphases and G2-PCCs of donor 1 was higher than donor 2 at all the radiation doses relative to metaphase chromosomes (Table 3 and Fig 5). Correlation coefficient analysis performed on comparative yields of total aberrations between colcemid arrested metaphases and calyculin A induced G2-PCCs revealed the r value of 0.99 for both donors.

To verify whether the differences observed in the yields of aberrations between G2-PCCs and colcemid arrested metaphase chromosomes in the two donor samples are statistically significant, different types of unstable chromosome aberrations were analyzed by making separate models for dicentrics, centric rings, acentric rings, compound fragments, interstitial fragments, and terminal fragments. These 6 different outcomes were correlated and modeled by a multinomial distribution. To compare the aberration yields between G2-PCCs and metaphase chromosomes, a variable called G2 indicator was created and coded as 0 for metaphase chromosomes and 1 for G2-PCCs. Dose response models with an intercept and beta component only by adding a modification of the intercept by donors and an interaction term between beta and G2 indicator were used. Alpha component of LQ was not included because of the fewer radiation doses used in this study. Our main purpose was to determine whether the interaction between beta component of LQ and G2 indicator for G2-PCC was statistically different from colcemid arrested metaphases for each of the outcomes (dicentrics, centric rings, acentric rings, compound, interstitial, and terminal fragments). The results revealed a significant difference (*p*-value 0.037) only for dicentrics/total aberrations (dicentrics, rings and fragments), which was somewhat lower for G2-PCCs relative to that of metaphase chromosomes. The *p*-values were > 0.05 for other aberration types (outcomes).

### Enrichment and estimation of absorbed radiation dose in calyculin A induced G2-PCCs prepared after 36 hrs and 48 hrs of culture

**Fig 6:**
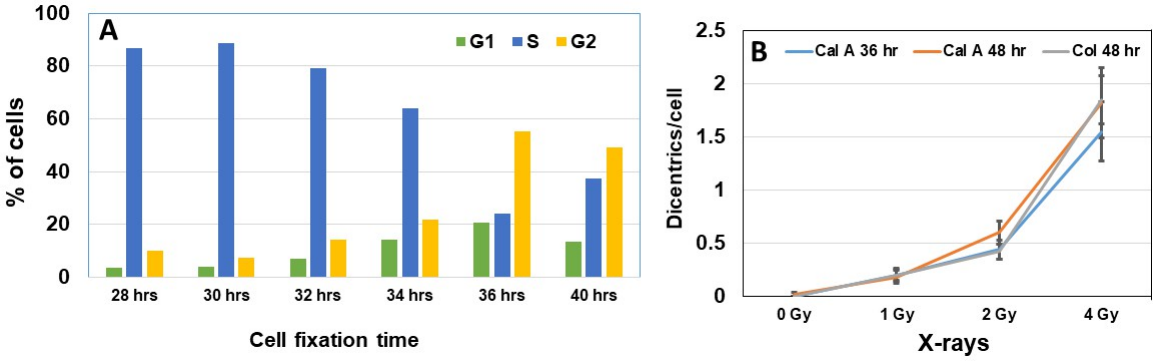
(A). Percentages of G2 cells detected in the lymphocyte cultures harvested at different times following treatment for 30 min with calyculin A. Cultures harvested at 36 hrs and 40 hrs showed the enrichment of cells with G2-PCCs. (B) Frequencies of dicentric chromosomes detected in calyculin A induced G2-PCCs after fixation of lymphocytes at 36 hrs and 48 hrs relative to conventional colcemid arrested metaphase chromosomes prepared from lymphocytes cultured for 48 hrs. Error bars indicate SEM.

The chemically induced G2-PCC assay is routinely performed after culturing the lymphocytes for 48 hrs. To test the feasibility of obtaining sufficient G2-PCCs at fixation times shorter than 48 hrs which would facilitate a rapid triage, lymphocytes were cultured for different times (28 hrs, 30 hrs, 32 hrs, 34 hrs, 36 hrs, and 40 hrs) followed by treatment with calyculin A for 30 min. Among the various culture times, lymphocytes grown for 36 hrs and 40 hrs yielded the maximum percentage of G2-PCCs (> 50%; Fig 6A).

Data obtained on the yields of dicentric chromosomes in G2-PCCs (obtained after 36 hrs and 48 hrs of culture) as well as colcemid arrested metaphase chromosomes (prepared from 48 hrs of culture) are given in Table 4 and Fig 6B. The frequency of dicentric chromosome per cell detected in the G2-PCCs after 36 hrs of culture was 0.20 ± 0.06 for 1 Gy, 0.44 ± 0.09 for 2 Gy and 1.55 ± 0.28 for 4 Gy while the frequency for the G2-PCCs after 48 hrs of culture was 0.18 ± 0.06 for 1 Gy, 0.60 ± 0.11 for 2 Gy and 1.82 ± 0.33 for 4 Gy. The dicentric chromosome frequency observed in the metaphase chromosomes after 48 hrs culture was essentially like G2-PCCs of 48 hrs for both 1 Gy and 4 Gy but was lower for 2 Gy (0.42 ± 0.07). Using a standard calibration curve generated in our laboratory based on the dicentric yields detected by FISH (Y = 0.008 ± 0.004 + 0.088 ± 0.022*D + 0.095 ± 0.009 *D^2^ where Y is the yield of dicentrics, and D is the radiation dose), absorbed radiation doses were estimated for both G2-PCCs (36 hrs and 48 hrs) and metaphase chromosomes (48 hrs of culture). The estimated doses with lower and upper limit at 95% confidence interval for G2-PCCS and metaphase chromosomes are shown in Table 5. Our results indicate that the FISH-based G2-PCC assay can be considerably shortened to 36 hrs for obtaining satisfactory dose estimates that are comparable to conventional G2-PCC assay and DCA that are performed for 48 hrs.

## Discussion

DCA has become the universally accepted technique for estimating the absorbed radiation dose in individuals exposed to occupational, incidental, and accidental exposures of different radiation qualities. Despite being the “gold standard” for absorbed dose estimation, the practical applicability of DCA to large-scale radiological/nuclear incidents is severely restricted due to its time consuming and labor-intensive attributes. Optimal lymphocyte proliferation is yet another crucial factor for DCA since dicentric chromosomes are detected at the metaphase stage of mitosis. Radiation exposures exceeding 5 Gy of photons greatly reduce the proliferation of lymphocytes and therefore estimation of absorbed radiation dose by DCA poses a challenge after acute high dose exposures. To overcome some of these technical limitations associated with conventional DCA, attempts were made to prematurely condense interphase chromosomes by DNA replication inactivated Sendai virus or polyethylene glycol (PEG) mediated cell fusion in G0/G1 and by treatment with protein phosphatase inhibitors in G2 stage of cell cycle [1–3, 5, 9]. Among the two phosphatase inhibitors, calyculin A has been demonstrated to be superior to okadaic acid in terms of reduced cytotoxicity and a much higher yield of G2-PCCs in human cells [5, 13].

The main purpose of this study is to evaluate the potential of FISH based G2-PCC assay as a triage biodosimetry tool for assessing a wide range of low and acute high radiation exposures of photons. Previous studies [12, 13, 15–19] utilized the Giemsa staining method to quantify IR induced aberrations in colcemid arrested metaphase cells. In the current study, FISH using centromere and telomere specific PNA probes was utilized to classify radiation induced unstable chromosome aberrations in G2-PCCs and to evaluate their suitability for rapid biodosimetry. One distinct advantage of using G2-PCC assay over the conventional DCA is that acute radiation exposures exceeding 5 Gy can be easily detected and the frequencies of any of the three unstable aberrations—dicentrics, rings and fragments—can be used for absorbed dose estimation once the calibration curves are established for each of the aberration types.

The G2-PCC-FISH assay we used in the current study revealed a dose dependent induction of dicentric and ring chromosomes up to 20 Gy of X-rays. Although the G2-PCC assay is superior to conventional DCA in terms of detecting high radiation dose exposures, the turnaround time for dose estimation is still 3–4 days for both assays. Nakayama et al. [21] recently demonstrated that enough calyculin A induced G2-PCCs could be obtained for chromosome analysis after 40 hrs of *ex vivo* culture of lymphocytes and that caffeine treatment further increased the proportion of G2-PCCs. The current study demonstrated that G2-PCCs obtained after 36 hrs of lymphocyte culture (mock and irradiated) yielded absorbed dose estimates that were comparable to G2-PCCs, and metaphase chromosomes obtained after 48 hrs of culture under triage mode of scoring (50 cells or 30 dicentrics). It has been generally accepted that the triage cut off dose is 2 Gy, and the estimated dose for the actual delivered dose of 2 Gy was 1.71 ± 0.18 Gy ((1.27–2.20 Gy, 95% CI) for G2-PCCs obtained after 36 hrs of culture and 1.66 ± 0.14 Gy (1.35–2 Gy, 95%CI) for metaphases obtained after 48 hrs of culture. Conventional DCA requires 48 hrs for lymphocyte culturing which creates a bottleneck when several hundreds of samples are to be processed for dose estimation. Results of our study suggest that G2-PCCs prepared after 36 hrs of culture can be effectively used for a rapid medical triage by considerably shortening the culture time by 12 hrs. Increased detection of chromosome aberrations in G2-PCCs by FISH over conventional Giemsa-stained metaphases is yet another advantage for improving the dose prediction accuracy especially for low dose exposures (0.1 Gy—0.5Gy) through the analysis of all types of unstable aberrations.

In this study, the frequency of ring chromosomes also exhibited a dose dependent increase up to 15 Gy with a slight decline at 20 Gy. Although the interphase chromosomes are prematurely condensed, they have been successfully utilized for the analysis of radiation induced chromosome aberrations. An earlier study [15] used calyculin A induced PCC rings and fragments for dose assessment after high dose exposures. Applicability of calyculin A induced hollow rings for biodosimetry was demonstrated in both G1 and G2 PCCs [16] and the formation of total and hollow rings followed a Poisson distribution with the dose effect relationship showing a linear fit. Utility of G2-PCC objects in dose assessment of ex vivo and in vivo irradiated human samples has been recently demonstrated [22]. Wang et al. [18] demonstrated the induction of G2 PCC fragments in human peripheral blood lymphocytes that were irradiated with 5–50 Gy of charged heavy ion particles. In this study, the dose effect relation for the induction of fragments was observed up to 30 Gy and the G2-PCCs were not detectable when the exposure dose exceeded 30 Gy. Observation of aberrations in human lymphocytes after high radiation dose exposures (5–30 Gy) indicate the possibility that G2-PCC assay can be effectively employed for scenarios involving high doses of partial body exposures in humans.

Comparison on the yields of IR induced unstable chromosome aberrations (dicentrics, rings and fragments) in colcemid arrested metaphase chromosomes and in calyculin A induced G2-PCCs has not been done in any of the previous studies. By performing a head-to-head comparison between metaphase chromosomes and G2-PCCs in the blood samples of two donors, we demonstrated higher frequencies of all the unstable aberrations in G2-PCCs, but these differences were not statistically significant. Among the aberrations, centric rings and compound fragments were induced predominantly relative to acentric rings, terminal, and interstitial fragments. All these unstable aberrations can be used either singly or in combination for dose estimation over a wide range of radiation dose exposures. It is of interest to note that the frequency of rings observed in donor 2 for 5 Gy and 7.5 Gy of X-rays was higher than donor 1, although the frequencies of dicentrics and fragments were less in donor 2. Future studies are required to determine whether inter-individual heterogeneity exists for the induction of rings. Utilization of FISH on calyculin A induced G2-PCCs not only detected increased frequencies of unstable chromosome aberrations but also enabled the categorization of rings and excess fragments based on the presence or absence of centromeres (rings) and telomeres (fragments). This information may be useful for quantifying the number of DNA double strand breaks that are either mis-rejoined or non-rejoined (terminal and interstitial fragments).

In our study, the excess chromosome fragments were induced by 1.6–1.8 folds higher than that of dicentrics in both colcemid arrested metaphase chromosomes and calyculin A induced G2-PCCs. A recent study utilized the excess fragments for the assessment of DNA damage induced by high doses of protons and photons [24]. Efforts are made in some of the biodosimetry laboratories in the USA to develop algorithm(s) for the automated detection of excess PCC fragments both in G0-PCCs and G2-PCCs. Application of the shortened version of G2-PCC assay as demonstrated in this study together with the automated detection of excess PCC fragments can serve as rapid triage tool. Although FISH technique can dramatically improve the resolution for detection of various types of unstable chromosome aberrations, many laboratories may not have the required resources such as the PNA probes, fluorescent microscope, and image acquisition/analysis software. Under those circumstances, analysis of excess PCC fragments for dose estimation after acquisition of images using transmitted light microscope can be useful. Currently, efforts are undergoing in our laboratory for optimizing the automated detection of dicentric chromosomes and G2 PCCs after the FISH staining of centromeres and telomeres. Once optimized, automated detection can considerably reduce the turnaround time for absorbed dose estimation. Since most, if not all, accidental and diagnostic overexposures in humans are non-uniform with extremely high radiation doses, calyculin A induced G2-PCC assay may prove to be superior to conventional DCA for the dose assessment of those acute high radiation exposures. Future studies employing a whole range of low, moderate, and high doses of different radiation qualities as well as simulated partial body exposures are required for validation of FISH based G2-PCC assay as an effective biodosimetry tool for triage.

## Conclusions

Calyculin A induced G2-PCC assay coupled with FISH staining has the capability to detect the whole spectrum of unstable chromosome aberrations induced by a wide range of low and acute high radiation dose exposures. The shortened version of G2-PCC demonstrated in this study will be useful as a rapid biodosimetry triage tool for large-scale radiological/nuclear incidents where several hundreds of individuals can get exposed to substantial doses of radiation.

## Supporting information

S1 Table Statistical analysis for the induction of unstable chromosome aberrations in metaphase chromosomes and G2-PCCs. Estimated values for Var/Mean ±SE and U for dicentrics, centric rings, acentric rings, and fragments after various doses of x-rays in the blood samples of two donors are shown for colcemid arrested metaphase chromosomes and calyculin A induced G2-PCCs.
(DOCX)
