# Supplementary material for: Application of FISH based G2-PCC assay for the cytogenetic assessment of high radiation dose exposures: Potential implications for rapid triage biodosimetry
Source: PLoS One. 2024 Oct 25;19(10):e0312564. doi: 10.1371/journal.pone.0312564 (PMC11508073; doi:10.1371/journal.pone.0312564)
Supplement: S1 Table — Estimated values for Var/Mean ±SE and U for dicentrics, centric rings, acentric rings, and fragments after various doses of x-rays in the blood samples of two donors are shown for colcemid arrested metaphase chromosomes and calyculin A induced G2-PCCs. (DOCX) [file pone.0312564.s001.docx]

**S1 Table. Statistical analysis for the induction of unstable chromosome aberrations in metaphase chromosomes and G2- PCCs**

**Metaphase chromosomes**

| Sample | Dose (Gy) | Var/Mean ± SE | U | Var/Mean  ± SE | U | Var/Mean  ± SE | U | Var/Mean  ± SE | U |
| --- | --- | --- | --- | --- | --- | --- | --- | --- | --- |
| Donor 1 | 0 | 0 | 0 | 0 | 0 | 0 | 0 | 0.99±0.07 | -0.11 |
|  | 0.5 | 0.92± 0.08 | -0.87 | 0 | 0 | 0 | 0 | 1.19±0.08 | 2.20 |
|  | 1 | 1.05± 0.08 | 0.54 | 0 | 0 | 0 | 0 | 1.19±0.08 | 2.18 |
|  | 3 | 0.85±0.10 | -1.44 | 0 | 0 | 0.98±0.08 | -0.17 | 0.89±0.10 | -1.07 |
|  | 5 | 1.10±0.14 | 0.68 | 0.97±0.12 | -0.24 | 1.37±0.13 | 2.75 | 1.13±0.14 | 0.92 |
|  | 7.5 | 0.72±0.14 | -1.91 | 0.96±0.12 | -0.31 | 1.14±0.13 | 0.99 | 0.92±0.14 | -0.53 |
| Donor 2 | 0 | 0 | 0 | 0 | 0 | 0 | 0 | 1.38±0.08 | 4.50 |
|  | 0.5 | 0.94±0.10 | -0.52 | 0 | 0 | 0 | 0 | 1.03±0.11 | 0.24 |
|  | 1 | 0.89±0.10 | -1.08 | 0 | 0 | 0.98±0.08 | -0.12 | 0.96±0.10 | -0.35 |
|  | 3 | 1.17±0.11 | 1.52 | 0.99±0.08 | -0.08 | 0.98±0.09 | -0.14 | 1.25±0.11 | 2.19 |
|  | 5 | 0.59±0.21 | -1.88 | 1.00±0.00 | 0 | 0.95±0.17 | -0.26 | 1.16±0.21 | 0.76 |
|  | 7.5 | 0.98±0.44 | -0.03 | 0 | 0 | 1.53±0.36 | 1.46 | 1.28±0.44 | 0.63 |

**Dicentrics Acentric rings Centric rings Fragments**

**G2-PCCs**

**Dicentrics Acentric rings Centric rings Fragments**

| Sample | Dose (Gy) | Var/Mean ± SE | U | Var/Mean  ± SE | U | Var/Mean  ± SE | U | Var/Mean  ± SE | U |
| --- | --- | --- | --- | --- | --- | --- | --- | --- | --- |
| Donor 1 | 0 | 0 | 0 | 0 | 0 | 0 | 0 | 1.05±0.08 | 0.62 |
|  | 0.5 | 1.10±0.08 | 1.12 | 0 | 0 | 0 | 0 | 2.60±0.08 | 18.00 |
|  | 1 | 0.96±0.08 | -0.46 | 0 | 0 | 0.99±0.07 | -0.11 | 1.57±0.08 | 6.39 |
|  | 3 | 1.09±0.10 | 0.87 | 1.13±0.09 | 1.39 | 0.95±0.09 | -0.52 | 1.32±0.10 | 3.20 |
|  | 5 | 0.87±0.14 | -0.90 | 0.94±0.13 | -0.38 | 1.01±0.13 | 0.09 | 0.91±0.14 | -0.56 |
|  | 7.5 | 0.68±0.14 | -2.19 | 0.92±0.13 | -0.53 | 0.94±0.13 | -0.43 | 0.86±0.14 | -0.92 |
| Donor 2 | 0 | 0 | 0 | 0 | 0 | 0 | 0 | 2.10±0.08 | 12.70 |
|  | 0.5 | 1.10±0.08 | 1.12 | 0 | 0 | 0 | 0 | 1.48±0.08 | 5.36 |
|  | 1 | 1.02±0.08 | 0.25 | 0.99±0.07 | -0.11 | 1.26±0.08 | 3.17 | 1.22±0.08 | 2.42 |
|  | 3 | 1.05±0.10 | 0.49 | 0.96±0.09 | -0.42 | 0.93±0.09 | -0.67 | 2.66±0.10 | 16.50 |
|  | 5 | 0.81±0.14 | -1.32 | 0.85±0.13 | -1.03 | 0.95±0.13 | -0.31 | 1.28±0.14 | 1.98 |
|  | 7.5 | 0.93±0.14 | -0.44 | 1.39±0.13 | 2.82 | 0.88±0.14 | -0.80 | 2.04±0.14 | 7.33 |
